# Changes of the perceived quality of care for older patients with hypertension by community health centers in shanghai

**DOI:** 10.1186/s12875-017-0683-4

**Published:** 2017-12-29

**Authors:** Haitao Li, Xiaolin Wei, Martin C. S. Wong

**Affiliations:** 1 School of Medicine, Shenzhen University, Shenzhen, China; 2 Division of Clinical Public Health and Institute of Health Policy, Management and Evaluation, Dalla Lana School of Public Health, University of Toronto, 155 College St, Toronto, ON M5T 3M7 Canada; 3 The Jockey Club School of Public Health and Primary Care, Faculty of Medicine, The Chinese University of Hong Kong, Hong Kong, SAR China

**Keywords:** Primary care, Quality of care, Hypertension, High blood pressure

## Abstract

**Background:**

Monitoring and evaluating changes of quality of primary care for older adult hypertensive patients is part of effective delivery of primary care. This study aimed to investigate changes of older adult hypertensive patients’ perceived quality of primary care over time in Shanghai.

**Methods:**

Two rounds of cross-sectional questionnaire surveys were conducted in Shanghai in November 2011 and June 2013. A total of 437 patients participated in the first Round survey and 443 in the second. Primary care attributes were collected from Community Health Center users through on-site face-to-face interview surveys using the validated Primary Care Assessment Tool. Multiple linear regressions were used to determine whether there was any difference in primary quality of care scores between 2011 and 2013 surveys.

**Results:**

Compared with those in the first Round, participants in the second Round reported higher scores in total primary care quality (28.73 vs. 27.75, *P* < 0.001), as well as primary care attributes including first-contact utilization (2.81 vs. 2.60, *P* < 0.001) and accessibility (2.48 vs. 2.44, *P* < 0.05), continuity of care (3.38 vs. 3.27, *P* < 0.001), coordination of information (3.82 vs. 3.67, *P* < 0.001), comprehensiveness of service availability (3.51 vs. 3.39, *P* < 0.001) and provision (2.69 vs. 2.43, *P* < 0.001), and cultural competence (2.67 vs. 2.49, *P* < 0.05), but a lower score in coordination of services (2.45 vs. 2.55, *P* < 0.05).

**Conclusion:**

Older adult hypertensive patients perceived better primary care quality from 2011 to 2013 in Shanghai. This may be associated with the general practitioner team service in Shanghai where hypertensive patients were targeted.

## Background

China is a rapidly aging society, where older population aged 60 years and above accounted for over 200 million in 2013 [1]. Hypertension is found to be the commonest chronic disease among this age group [2]. An estimated hypertension prevalence among Chinese aged from 60 to 69 years onwards was more than 50% [3]. Hypertension prevalence keeps rising in old age [3, 4]. However, hypertension control remains sub-optimal worldwide [5, 6], including China [3, 7, 8]. Therefore, understanding quality of care for older adult hypertensive patients has important implications for policy makers.

Key features of primary care quality include accessibility, continuity, coordination and comprehensiveness and patient centered care [9]. Accessibility has been found positively correlated with population health. Higher accessibility has also been shown leading to reduced socioeconomic and racial disparities in health. Coordination of care has been found have mixed results with respect to health. Patient satisfaction is correlated to certain features of the coordination of care. A strong association between continuity of care and improved patient satisfaction has been established. It was also found that continuity of care is cost-effective in primary care, and ensures greater efficiency of services. There is also a strong relevance of continuity of care to decreased hospitalization and improved early diagnosis. The delivery of a wide range of primary care services is associated with improved health outcomes [10]. Researchers have demonstrated the relevance of accessibility, continuity, coordination and comprehensiveness to the quality of management of hypertension [11].

In urban China, Community Health Centers (CHCs) are the major primary care providers to residents. Shanghai is a pioneer in exploring and developing CHCs in China. Early-built CHCs in Shanghai are transformed from first-level hospitals, while newly-built ones are established directly by the government [12]. By the end of 2010, there were 227 CHCs in Shanghai. These CHCs act as independent entities, funded fully and managed directly by the district government, rendering them not-for-profit [13].

Chronic disease management, including hypertension and diabetes, is one of the six integrated health services provided by CHCs since the launch of the primary care program in 1997. The Health Care Reform Plan Policy launched by the State Council of China in 2009 re-emphasized the relevance of the primary care approach in managing chronic diseases including hypertension [14]. Hypertensive patients can be detected through screening of health records or free health examination. An alternative for detection of hypertension is through mandatory BP tests of all patients over 35 years old in CHCs. All diagnosed hypertensive patients are registered and managed in their CHCs. CHCs are responsible for establishing a separate health record for standard management of hypertension, including regular monitoring of BP and complications, lifestyle modification advises, medication refill etc. The work is provided by a team led by the general practitioners, with doctors trained in Traditional Chinese Medicine, nurses and public health doctors. These strategies may impact the utilization of primary care by older hypertensive patients.

From 2011, residents are promoted to enroll in the general practitioner team to manage their health [15]. The team provides a free package of clinical and preventive care, including hypertension management. They, together with community organizations, also provide health education such as healthy diet and smoking cessation, which may improve comprehensiveness of service in CHCs. Capitation payment and pay-for-performance have been introduced [16]. Performance is determined by volume of services provided, their quality and patient satisfaction. The older adult hypertensive patients are thus easier to obtain needed care for better hypertension management (i.e., improved accessibility and continuity of care) [16].

Monitoring and evaluating changes of quality of primary care for older patients with hypertension is part of assurance of effective primary care delivery, and in maximizing its full potential. This study aimed to investigate changes in primary care quality perceived by older adult hypertensive patients from 2011 to 2013 in Shanghai. It also examined whether household income and multi-morbidity had any effect on perceived primary care quality in 2011 and 2013.

## Methods

### Ethics approval

Ethical approval was obtained from the Joint Chinese University of Hong Kong and New Territories East Cluster Clinical Research Ethnics Committee (Ref. No. CRE-2010.441). Shanghai Association of Community Health Care (SACH) reviewed the ethical point and approved it in 2011 until 2014 (SACH201103) (the whole study period).

### Participants and procedures

Two rounds of cross-sectional surveys were conducted in Shanghai in November 2011 and June 2013, respectively. Multistage stratified random sampling method was used to select CHCs as study settings. Firstly, four districts, namely Jing’an, Changning, Xuhui and Pudong, were selected randomly from four geographic areas. Secondly, one CHC was selected at random from each district, arriving at a total of 4 CHCs. Both rounds of surveys were performed in the same CHCs.

The sampling frame was the users’ population in the CHCs, with a planned sample size of 614. The sample size was estimated for additional planned analyses which is not the focus of this paper, but further information can be found in our previous publication [16]. Eight hundred subjects were intended to be interviewed when considering a 90% response rate and an 85% of usual CHC users. The inclusion criteria of the participants consisted of subjects who were: 1) age ≥ 18 years; 2) able to communicate and give informed consent; and 3) paid ≥1 CHC visit before the survey. Employing systematic sampling methods, at least 200 participants were recruited from each CHC in each round of the survey. The samples between the two rounds of surveys were different. Extensively trained interviewers performed on-site face-to-face interview surveys. Written informed consent from CHCs and participants was obtained before the surveys were commenced. In total, 811 and 795 participants completed the survey in Rounds 1 and 2 respectively, with response rates of 94% and 92%. We selected the subset for older hypertensive patients as this is the core group of patients managed in CHCs. There was a total of 437 and 443 participants were aged ≥60 years and had physician-diagnosed hypertension. This study was a pre-specified sub-analysis where the full dataset was reported elsewhere [16].

### Key measures

Primary Care Assessment Tool (PCAT) [17], which was translated, adapted and validated at Chinese primary care settings [18, 19], was used to measure quality of primary care for older patients with hypertension. The questionnaire asked about primary care attributes which were recognized as the structural bases of primary care process. These attributes included first contact utilization, first contact accessibility, continuity of care, coordination of services, coordination of information, comprehensiveness of service availability, comprehensiveness of service provision, patient focused care, community oriented and cultural competence. The answers to each question were based on a four-point Likert-type scale (definitely, probably, probably not, definitely not). Scores were calculated for each question, with a higher score indicating a better quality. Attributes scores were calculated as a mean score for all questions under the subject heading (range, 1–4). A total score was calculated by adding the scores for each attribute (range, 10–40). PCAT has been used as a tool for measuring changes in primary care quality over time [20, 21].

Socioeconomic and demographic characteristics of study subjects were collected in the survey including gender, age, self-reported health status, hypertension related co-morbidities, registration status, education level, occupation, monthly household income and health insurance. Health care utilization measures were also collected by asking two questions: 1) number of CHC visits during the past one year; and 2) length of time with the visited CHC since their first visit.

### Statistical analysis

The differences in individual and total primary care attributes scores between the two rounds of surveys were examined first by using independent two samples *t*-tests and then by multiple linear regression models after controlling possible confounders. We compared individual and total primary care attributes scores among the participants with different household income levels, and between the participants with and without multi-morbidities in each round of survey. Interaction between variables was also tested by including an appropriate term in the model. Potential confounders in the multiple linear regression models included socio-demographic characteristics (i.e., gender, self-reported health status, presence of multi-morbidities, registration status, education level, occupation, household income, health insurance) and health care utilization measures (number of CHC visits and length of time with the CHC). The assumptions of multiple linear regression models such as linearity, independence of errors, equal variance of errors, as well as normality of errors were tested. Model fittings were conducted by using backward elimination with a threshold of 0.10 for variable inclusion in the model. For all tests conducted in the study, a *P* value of <0.05 was regarded as statistically significant. All analyses were performed using SPSS20.0.

## Results

No significant differences were identified in terms of gender, self-reported health status, registration status, education level, occupation, health insurance, as well as number of CHC visits between study participants in the two rounds. However, compared with the participants in the Round 1, the subjects in the Round 2 were less likely to have multi-morbidities (63.8% vs. 50.3%, *P* < 0.001). The participants in the Round 2 tended to have a higher income than those in the Round 1 (*P* < 0.001). The participants in the Round 2 had been utilizing services provided by the CHCs for a longer period when compared with those in the Round 1 (*P* = 0.002) (Table 1).

**Table 1:** Socioeconomic and demographic characteristics, and health care utilization measures of the older patients with hypertension in Round 1 and Round 2 surveys

Characteristics	Round 1(*N* = 437) n(%)	Round 2(*N* = 443) n(%)	*P* value
*Demographics*			
Gender			0.618
Female	293(67.0)	289(65.2)	
Male	144(33.0)	154(34.8)	
Self reported health status			0.450
Good and above	75(17.2)	84(19.0)	
Fair	283(65.8)	292(65.9)	
Poor	79(18.1)	67(15.1)	
Presence of multi-morbidities^a^			*<0.001*
Yes	279(63.8)	223(50.3)	
No	158(36.2)	220(49.7)	
*Socioeconomics*			
Registration^b^			1.000
Locals	421(96.3)	426(96.2)	
Migrants	16(3.7)	17(3.8)	
Education			0.120
5-year university and above	46(10.5)	65(14.7)	
3-year college	45(10.3)	55(12.4)	
High school and equivalent	126(28.8)	134(30.2)	
Middle school	154(35.2)	126(28.4)	
Primary school and below	66(15.1)	63(14.2)	
Occupation^c^			0.298
Have a job	5(1.1)	10(2.3)	
Do not have a job	432(98.9)	433(97.7)	
Household income^d^			*<0.001*
Below poverty line	75(17.2)	48(10.8)	
Between poverty line and median	328(75.1)	323(72.9)	
Above median	34(7.8)	72(16.3)	
Health insurance^e^			0.093
Yes	432(98.9)	430(97.1)	
No	5(1.1)	13(2.9)	
*Visits to the primary care provider*			
Number of visits in the last year, n(%)			0.167
≤ 12 visits	94(21.5)	110(24.8)	
13–24 visits	88(20.1)	101(22.8)	
25–36 visits	68(15.6)	75(16.9)	
≥ 37 visits	187(42.8)	157(35.4)	
Length of time with the health facility			*0.002*
≤ 1 year	25(5.7)	16(3.6)	
1~2 years	49(11.2)	23(5.2)	
3~4 years	55(12.6)	49(11.1)	
≥ 5 years	308(70.5)	355(80.1)	

^a^Hypertension related co-morbidities included cerebrovascular disease, diabetes, heart disease, kidney disease and high cholesterol

^b^Registration status dividedthe participants into two groups – migrants and locals – based on their *hukou* status, which was usually used to identify a person’s official place of residence [28]

^c^The participants were classified into two groups, those who had a job (including employed and self-employed) and those who did not have a job (including the retired, students, unemployed and housewives)

^d^The participants were allocatedinto three economic groups according to monthly household poverty line (RMB3,000/US$484) and mean household income level (RMB10,000/US$1282) in 2011 [30]

^e^The participants with health insurance referred to those covered by local health insurance schemes including Basic Medical Insurance Scheme for Urban Employees and Basic Medical Insurance Scheme for Urban Residents

Participants in the Round 2 reported a higher score in first contact utilization (2.81 vs. 2.60, *P* < 0.001), continuity of care (3.38 vs. 3.27, *P* < 0.001), coordination of information (3.82 vs. 3.67, *P* < 0.001), comprehensiveness of service availability (3.51 vs. 3.39, *P* < 0.001) and provision (2.69 vs. 2.43, *P* < 0.001), as well as cultural competence (2.67 vs. 2.49, *P* < 0.05), while a lower score in coordination of services (2.45 vs. 2.55, *P* < 0.05) when compared with those in the Round 1. The total score reported by participants in the Round 2 was also higher than that in the Round 1, 28.73 and 27.75 respectively (*P* < 0.001). These significant differences still remained after controlling for confounding variables. Additionally, multiple regression analysis identified a significantly higher score among participants in Round 2 when compared with those in Round 1 with respect to first contact accessibility (2.48 vs. 2.44; β = 0.045, 95% CI: 0.002–0.088) (Table 2).

**Table 2:** Individual and total primary care attributes scores reported by the older patients with hypertension in Round 1 and Round 2 surveys

Attributes	Round 1(SE) (*N* = 437)	Round 2(SE)^a^ (*N* = 443)	Adjusted β(95% CI)^b^
First contact-utilization	2.60(0.028)	2.81(0.026)^***^	0.206(0.132,0.280)^***^
First contact-accessibility	2.44(0.014)	2.48(0.016)	0.045(0.002,0.088)^*^
Continuity of care	3.27(0.020)	3.38(0.021)^***^	0.132(0.076,0.188)^***^
Coordination of services	2.55(0.026)	2.45(0.030)^*^	−0.105(−0.184,-0.025)^*^
Coordination of information	3.67(0.021)	3.82(0.018)^***^	0.147(0.090,0.204)^***^
Comprehensiveness- service availability	3.39(0.019)	3.51(0.015)^***^	0.092(0.044,0.139)^***^
Comprehensiveness- service provided	2.43(0.028)	2.69(0.027)^***^	0.260(0.182,0.339)^***^
Family centeredness	2.79(0.040)	2.73(0.040)	−0.059(−0.172,0.053)
Community orientation	2.11(0.033)	2.18(0.037)	0.070(−0.029,0.170)
Cultural competence	2.49(0.051)	2.67(0.052)^*^	0.181(0.035,0.328))^*^
Total score	27.75(0.172)	28.73(0.166)^***^	0.969(0.501,1.438)^***^

*SE* Standard error, *CI* Confidence level;

^a^Independent two-sample *t*-test,

^b^Multiple linear regression analysis where dependent variables were individual and total primary care attributes scores, while covariates were gender, self-reported health status, presence of multi-morbidities, registration, education, occupation, household income, health insurance, number of CHC visits and length of time with the CHC;

β: Calculated with Round 1 as the reference;

^*^ *P* < 0.05;

***P < 0.001

In the Round 1, compared with the participants having a higher household income level, those in the low income group reported a higher score in first contact utilization (*P* < 0.05), while lower scores in continuity of care (*P* < 0.05) and comprehensiveness of service availability (*P* < 0.05). In the Round 2, the participants in the higher income group than in the lower income group were found to have a higher score regarding comprehensiveness of service availability (*P* < 0.05) and family centeredness (*P* < 0.05), but a lower score in coordination of information (*P* < 0.05). However, no significant difference was identified in total primary care quality score among the participants with different household income levels in both rounds of surveys (Table 3).

**Table 3:** Individual and total primary care attributes scores reported by the older patients with hypertension with different household income levels in the two rounds of surveys

Attributes	Round 1, mean(SE)^a^			Round 2, mean(SE)^a^		
	Low(*N* = 75)	Middle(*N* = 328)	High(*N* = 34)	Low(*N* = 48)	Middle(*N* = 323)	High(*N* = 72)
First contact-utilization	2.74(0.064)	2.58(0.032)*	2.51(0.104)*	2.83(0.081)	2.80(0.031)	2.84(0.062)
First contact-accessibility	2.46(0.033)	2.43(0.016)	2.48(0.057)	2.47(0.049)	2.50(0.019)	2.44(0.039)
Continuity of care	3.23(0.049)	3.27(0.023)	3.37(0.067)*	3.33(0.078)	3.40(0.023)	3.28(0.061)
Coordination of services	2.56(0.055)	2.56(0.030)	2.47(0.092)	2.55(0.090)	2.43(0.037)	2.51(0.066)
Coordination of information	3.62(0.059)	3.68(0.024)	3.74(0.066)	3.89(0.038)	3.83(0.020)	3.73(0.064)*
Comprehensiveness- service availability	3.29(0.052)	3.41(0.021)*	3.41(0.076)*	3.43(0.038)	3.52(0.018)*	3.52(0.034)*
Comprehensiveness-service provided	2.47(0.067)	2.43(0.032)	2.36(0.105)	2.64(0.085)	2.68(0.032)	2.77(0.072)
Family centeredness	2.64(0.086)	2.82(0.047)	2.84(0.134)	2.58(0.116)	2.71(0.047)	2.92(0.099)*
Community orientation	2.25(0.077)	2.07(0.038)	2.14(0.117)	2.09(0.116)	2.17(0.042)	2.30(0.106)
Cultural competence	2.48(0.108)	2.47(0.061)	2.61(0.173)	2.79(0.151)	2.63(0.061)	2.76(0.135)
Total score	27.75(0.391)	27.73(0.204)	27.94(0.562)	28.59(0.525)	28.67(0.186)	29.09(0.472)

*SE* Standard error;

^a^Multiple linear regression analysis where dependent variables were individual and total primary care attributes scores, while independent variables were gender, self-reported health status, presence of multi-morbidities, registration, education, occupation, household income, health insurance, number of CHC visits and length of time with the CHC;

Respondents with low income were used as reference group;

*P < 0.05

In the first round of survey, there were no significant differences in all individual and total primary care attributes scores between the participants with and without multi-morbidities. However, in the second round of survey, the participants with multi-morbidities reported a higher score in coordination of services (2.47 vs. 2.44, *P* < 0.001) and coordination of information (3.84 vs. 3.80, *P* < 0.001) when compared with those without multi-morbidities (Table 4).

**Table 4:** Individual and total primary care attributes scores reported by the older patients with hypertension with and without multi-morbidities in the two rounds of surveys

Attributes	Round 1, mean(SE)^a^		Round 2, mean(SE)^a^	
	Without(*N* = 158)	With(*N* = 279)	Without(*N* = 220)	With(*N* = 223)
First contact-utilization	2.59(0.047)	2.60(0.034)	2.84(0.036)	2.78(0.038)
First contact-accessibility	2.44(0.024)	2.44(0.018)	2.47(0.023)	2.49(0.023)
Continuity of care	3.23(0.034)	3.29(0.024)	3.41(0.030)	3.35(0.030)
Coordination of services	2.59(0.041)	2.54(0.033)	2.44(0.044)	2.47(0.042)*
Coordination of information	3.71(0.033)	3.65(0.027)	3.80(0.028)	3.84(0.024)*
Comprehensiveness- service availability	3.40(0.034)	3.39(0.023)	3.50(0.019)	3.51(0.023)
Comprehensiveness-service provided	2.39(0.049)	2.46(0.034)	2.70(0.039)	2.67(0.039)
Family centeredness	2.84(0.067)	2.76(0.050)	2.78(0.058)	2.69(0.055)
Community orientation	2.11(0.058)	2.11(0.040)	2.21(0.053)	2.16(0.053)
Cultural competence	2.57(0.088)	2.44(0.063)	2.66(0.075)	2.69(0.072)
Total score	27.86(0.297)	27.69(0.212)	28.81(0.238)	28.65(0.230)

SE: Standard error;

^a^Multiple linear regression analysis where dependent variables were individual and total primary care attributes scores, while independent variables were gender, self-reported health status, presence of multi-morbidities, registration, education, occupation, household income, health insurance, number of CHC visits and length of time with the CHC;

Respondents without multi-morbidities were used as reference group;

*P < 0.05

## Discussion

This study in Shanghai found that total primary care quality perceived by older patients with hypertension improved significantly from 2011 to 2013, as reflected in improved total scores of care quality and its key attributes such as first contact utilization and accessibility, continuity of care, coordination of information, comprehensiveness of service availability and provision, and cultural competence. However, coordination of services score was lower in 2013compared to that in 2011. Although no significant difference was identified in total primary care quality score among the participants with different household income levels in each round of survey, there was some association between lower household income and lower scores in specific PCAT domains.

Increased scores in first contact suggests an improved gate-keeping role played by CHC provided primary care. This may be linked to improved patient confidence of CHC doctors due to its better capacity through training [22]. Improved scores in first contact accessibility and continuity of care echoed a previous study of all primary care users in Shanghai [16]. From 2011, older adult hypertensive patients are promoted to enroll in the general practitioner team service in CHCs [15]. The enrollees can receive hypertension management and elderly care free of charge. A free annual risk assessment and a longer duration per prescription, from two weeks to up to eight weeks, are also the benefits to enrollees. Capitation payment and pay-for-performance have been introduced [16]. Performance is determined by volume of services provided, their quality and patient satisfaction. The older adult hypertensive patients are thus easier to obtain needed care for better hypertension management (i.e., improved accessibility) [16]. The strategy encourages CHC doctors to improve continuity of care for improved performance. Studies showed improved overall care scores in Shanghai as compared with Shenzhen [16] and Kunming [23] where the general practitioner team policy was not or partially implemented.

We found improved score in coordination of information, but decreased score in coordination of services. The former may be linked with the establishment of a single medical insurance card that contains all medical consultations in Shanghai, which may help CHC doctors to recognize older adult hypertensive patients’ multi-morbidities and their past medical history [16, 24]. The latter may be related to the relatively slow progress regarding the referral system between public hospitals and CHCs. This may be due to the competition among medical institutions for patients and profits and the lack of incentives from public hospitals to collaborate with CHCs [25].

We reported improved scores in comprehensiveness of service availability and provision, suggesting that it is more likely for older adult hypertensive patients to receive non-pharmacological and pharmacological treatments to control the disease. Primary care provider team in CHCs provides a free package of clinical and preventive care for the enrolled older adult hypertensive patients. They, together with community organizations, also provide health education such as healthy diet and smoking cessation, which may improve comprehensiveness of service availability and provision. A previous study showed that hypertensive patients in Shanghai received more pharmaceutical care compared with those in Shenzhen [12]. Life-style modification initiatives are complementary to pharmaceutical treatment, and needs to be incorporated at the point of care delivery [26].

Our study did not find significant differences in total perceived primary care quality among the older adult hypertensive patients with different household income levels in both Round 1 and Round 2, which are consistent with the findings of previous studies [16, 27]. However, we did found that patients with lower household income reported lower scores for first contact utilization, continuity of care, comprehensiveness availability and family centeredness. This may reflect the inequalities of primary care quality in Shanghai primary care settings. From the health system point of view, Shanghai’s primary care is fully publicly owned and operated, and showed less inequality among different income groups compared with Shenzhen, where CHCs are hospital-owned and privately operated [16]. Other studies showed that Shanghai’s primary care is more equitable compared with that of Hong Kong, where public service only account for 20%, [22, 25] and Shenzhen, where a large proportion of its users, such as migrants, were not entitled to health insurance schemes [28]. Perceived quality of primary care tends to be equitable in public and no-profit primary care oriented systems such as UK, but not in countries with a large for-profit private sector in primary care [29].

The limitations of the study should be mentioned. Firstly, the generalizability of findings is limited since only 4 out of more than 200 CHCs were selected as study settings which may introduce sampling bias. Secondly, the participants were CHC users and were selected based on random cluster sampling methods, which prevents the findings from extending to the general older hypertensive patients who did not utilize services in CHCs. Thirdly, the patient-reported PCAT scores may be subject to recall bias. Finally, since this study compared findings between two cross-sectional surveys but not followed the subjects longitudinally, cause-and-effect relationship might not be directly ascertained.

## Conclusions

We found that older adult hypertensive patients in Shanghai had better experiences of primary care over time, especially with respect to first contact utilization and accessibility, continuity of care, coordination of information, comprehensiveness of service availability and provision, as well as cultural competence. Our findings on better perceived quality of primary care from 2011 to 2013 may be associated with the introduction of the general practitioner team service in Shanghai since 2011.

## Abbreviations

B: Blood pressure
CHCs: Community Health Centers
PCAT: Primary Care Assessment Tool
